# Decrease in pelvic incidence after adult spinal deformity surgery is a predictive factor for progression of hip joint osteoarthritis

**DOI:** 10.1186/s12891-024-07625-5

**Published:** 2024-06-28

**Authors:** Kazuo Tomizawa, Satoshi Inami, Hiroshi Moridaira, Haruki Ueda, Iwao Sekimoto, Tomoya Kanto, Hiroshi Taneichi

**Affiliations:** Department of Orthopaedic Surgery, Dokkyo Medical University, 880 Kitakobayashi, Mibu-Machi, Shimotuga-Gun, Tochigi, 321-0293 Japan

**Keywords:** Adult spinal deformity, Hip joint, Hip osteoarthritis, Pelvic incidence, Sagittal alignment

## Abstract

**Background:**

This study aimed to evaluate the association between spinopelvic alignment parameters and hip osteoarthritis progression after spinal alignment correction surgery for adult spinal deformity, focusing on the preoperative to postoperative change in spinopelvic alignment.

**Methods:**

This retrospective study enrolled 100 adult spinal deformity patients (196 hip joints) who underwent spinal fusion surgery, after excluding four joints with previous total hip arthroplasty. Acetabular roof obliquity (ARO), center edge angle (CE) and Kellgren and Lawrence (KL) grade were measured in the hip joint. Spinopelvic alignment parameters were measured preoperatively and 1-month postoperatively and the changes (Δ) during this period were calculated. Patients were followed-up for ≥ 5 years and factors associated with KL grade progression at 5-years postoperatively were determined by logistic regression analysis.

**Results:**

In the analysis with all cases, KL grade progressed in 23 joints. Logistic regression analysis revealed age (OR: 1.098, 95% CI: 1.007–1.198, *p* = 0.019), ARO (OR: 1.176, 95% CI: 1.01–1.37, *p* = 0.026), and Δ PI (OR: 0.791, 95% CI: 0.688–0.997, *p* < 0.001) as parameters significantly associated with KL grade progression. On the other hand, in the analysis limited to 185 cases with 1-month postoperative KL grade of 0, KL grade progressed in 13 joints. Logistic regression analysis revealed PI-LL (OR: 1.058, 95% CI: 1.001–1.117, *p* = 0.04), ΔPI (OR: 0.785, 95% CI: 0.649–0.951, *p* < 0.001), and ΔCobb (OR: 1.127, 95% CI: 1.012–1.253, *p* = 0.009) as parameters significantly associated with progression.

**Conclusions:**

Both the overall and limited analyzes of this study identified preoperative to postoperative change in PI as parameters affecting the hip osteoarthritis progression after spinal fusion surgery. Decrease in PI might represent preexisting sacroiliac joint laxity. Patients with this risk factor should be carefully followed for possible hip osteoarthritis progression.

## Background

Recent advances in surgical treatment for adult spinal deformity (ASD) have resulted in relief of low back pain and many disabilities caused by sagittal and coronal imbalance in many patients. However, correction of spinal malalignment requires spinal fusion, which sacrifices spinal segment mobility and alters spinopelvic biomechanics. Adjacent segment disease, which occasionally occurs at both proximal and distal junctions of the fused segment, are reported as an adverse effect of spinal fusion surgery [1–4].

Long spinal fusion surgery limits physiological motion of the pelvis, and a previous biomechanical study demonstrated that spinal fusion might increase mechanical stress in the hip joint [5]. Recent studies reported progression of hip osteoarthritis as adjacent segment disease after spinal fusion surgery. Kawai et al. reported that longer spinal fusion was associated with progression of hip joint narrowing following spinal fusion, although lumbosacral fusion was not a significant factor for joint narrowing [6]. Conversely, Kozaki et al. reported that spinal fusion surgery, including sacroiliac joint fixation, was a predictor of progression of hip joint narrowing [3].

The goal of alignment correction surgery for ASD is usually to increase lumbar lordosis (LL) to match pelvic incidence (PI) and to rotate a retroverted pelvis forwards, which decreases pelvic tilt (PT) and increases sacral slope (SS) [7]. These changes in pelvic position affect changes in the mechanical force on the hip joint [8]. Increased anterior coverage of the femoral head by the acetabulum might be a risk factor for femoroacetabular anterior impingement, which is considered a cause of hip osteoarthritis [9, 10]. However, the association between the change in position of the pelvis and hip osteoarthritis progression in postoperative ASD patients is unclear, because no previous studies have assessed preoperative to postoperative changes in spinopelvic alignment as a risk factor for hip osteoarthritis progression.

The purpose of this study was to evaluate the association between spinopelvic alignment and progression of hip joint osteoarthritis after spinal alignment correction surgery for ASD, focusing on the preoperative to postoperative spinopelvic alignment change.

## Methods

**Fig. 1:**
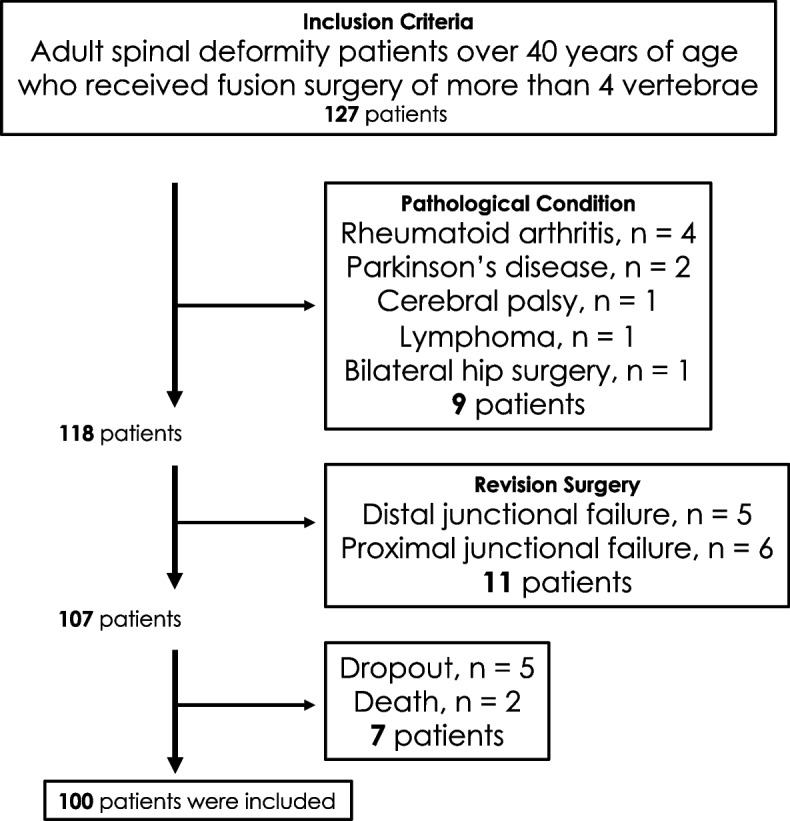
Flowchart showing patient inclusion and exclusion

This was a single-institutional, retrospective study of consecutive patients undergoing surgery for ASD between the years 2008 and 2015. Inclusion criteria were patients older than 40 years who underwent spinal fusion surgery for ASD of more than four vertebrae, available complete coronal and sagittal radiography from preoperative visits to final follow-up. Exclusion criteria were a presence of systemic inflammatory disease, neuromuscular disease, spinal tumor, and a history of bilateral hip surgery. Patients who underwent revision surgery due to junctional failure after primary surgery were also excluded (Fig. 1).

**Fig. 2:**
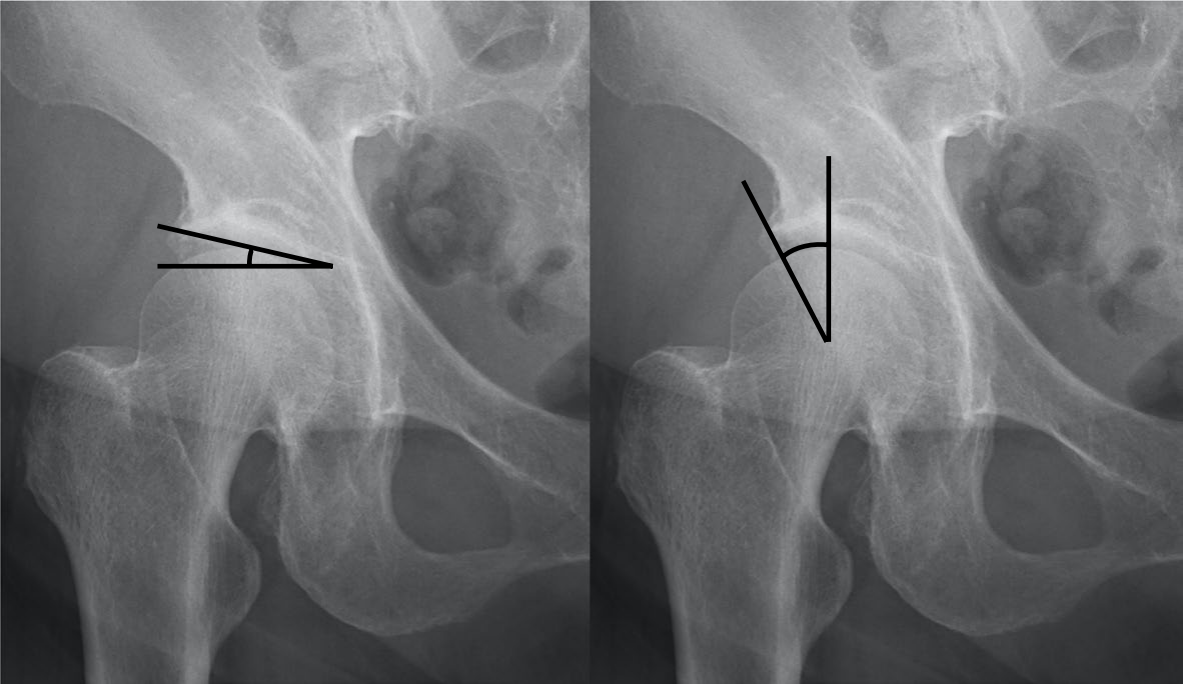
Measurements of hip joint parameters. Acetabular roof obliquity (ARO): the angle between the line connecting the inner edge and outer edge of the acetabular arch and the horizontal line passing the inner edge of the acetabular arch (left). Center edge angle (CE angle): the angle between the line connecting the femoral head center and lateral edge of the acetabulum and the vertical line (right)

All patients were followed-up for at least 5 years (mean follow-up period of 65.3 months (SD 3.2 months)). Preoperative and 1-month postoperative follow-up radiographic parameters were measured on full-length standing coronal and sagittal radiographs with the patients’ fingers on the clavicles and shoulders in 45° of forward elevation. Radiographic parameters of the spine that were evaluated included T5-T12 thoracic kyphosis (TK), T12-S1 LL, PT, SS, PI, sagittal vertical axis (SVA), PI minus LL (PI-LL), and Cobb angle of lumbar scoliosis. Although there were right convex and left convex coronal curves, the absolute value of the Cobb angle was recorded and analyzed. Radiographic parameters of the hip joint included acetabular roof obliquity (ARO) and center edge angle (CE) (Fig. 2).

Hip osteoarthritis was evaluated as the Kellgren and Lawrence grade (KL grade; grade 0: no osteoarthritis, grade 1: doubtful, grade 2: mild, grade 3: moderate, grade 4: severe) [11] at 1 month and 5 years postoperatively. This evaluation was done twice by two experienced hip surgeons. The matching coefficient kappa (rater 1 vs rater 2) for measurements of postoperative 1 month and 5 years were 0.76 and 0.78, respectively; reproducibility between the two independent observers was determined to be good. The kappa statics (first vs second measurement) for rater 1 of postoperative 1 month and 5 years were 0.85 and 0.87, respectively. Those for rater 2 of postoperative 1 month and 5 years were 0.85 and 0.8, respectively. Reproducibility between their first and second measurement was determined to be excellent. Discrepant cases were classified finally after consultation. Osteoarthritis progression was defined as an increase in KL grade at 5 years postoperatively compared to 1 month postoperatively.

Changes in the parameters (Δ) from preoperatively to 1 month postoperatively were also calculated. All radiographic measurements were conducted using measurement software (Centricity™ Enterprise Web, version 3.0, GE Healthcare Japan, Tokyo, Japan). This study was conducted with institutional review board approval, patient consent for study participation, and in accordance with the principles of the Declaration of Helsinki.

### Statistical analysis

Statistical analyses were performed using the JMP software package (JMP 14.2, SAS, Cary, NC). We divided all joints into 2 groups based on osteoarthritis progression, compared the data between the groups, and termed this analysis the overall analysis. Furthermore, in order to exclude the influence of pre-existing hip osteoarthritis on osteoarthritis progression, we excluded cases with KL grade 1–3 at 1 month postoperatively and performed an analysis limited to 185 cases with KL grade 0, and termed this analysis the limited analysis. The Wilcoxon test was used to compare continuous data. Chi-squared test was used for categorical outcomes. Logistic regression analysis using the parameters which had significant difference in the comparison studies as explanatory variables was performed to determine the factors associated with hip osteoarthritis progression. The level of significance was set at 0.05.

## Results

### Patient data

One-hundred patients (83 females) with a mean age of 65.8 years (SD 9 years; range 41–82 years) and a mean BMI 22.6% (SD 3.5%; range 14.5–35.6%), and 196 hip joints were included, after excluding four joints with previous total hip arthroplasty (THA).

The most common upper instrumented vertebrae were T10 (*n* = 52), followed by T11 and L2 (*n* = 9 each), T9 (*n* = 8), T12 (*n* = 7), L1 (*n* = 6), T8 (*n* = 3), T5 and L3 (*n* = 2 each), T4 and T7 (*n* = 1 each). The most common lower instrumented vertebrae were S1 (*n* = 76: including 68 sacroiliac joint fixations by iliac screws), followed by L5 (*n* = 13), L4 (*n* = 7) and L3 (*n* = 4). The mean number of fixation segments was 7.2 ± 2.1 (range: 3–14).

### Preoperative versus postoperative radiographic findings

The deformity correction surgery resulted in the preoperative deformity achieving approximately the physiological range at 1 month postoperatively (Table 1).

**Table 1:** Preoperative versus postoperative values of radiographic parameters

	Preoperative (Mean ± SD)	1 month postoperative (Mean ± SD)	Δ (Mean ± SD)
TK (˚)	16.6 ± 16.5	27.4 ± 12.3	10.8 ± 11
LL (˚)	6.3 ± 21.1	41.1 ± 11.9	34.8 ± 19.7
PT (˚)	34.4 ± 12.4	22.9 ± 9.3	-11.5 ± 9.2
SS (˚)	16.3 ± 11.1	27.5 ± 7.8	11.2 ± 8.7
PI (˚)	50.7 ± 10.9	50.4 ± 10.7	-0.4 ± 4.7
PI—LL (˚)	44.5 ± 22.6	9.2 ± 12.9	-35.3 ± 20.2
Cobb (˚)	28 ± 17.2	13.5 ± 10	-15 ± 11.7
SVA (mm)	106.7 ± 70	26.5 ± 35.1	-81.3 ± 67.6
ARO (˚)	8 ± 5.3	7.8 ± 5.4	0.3 ± 4.7
CE (˚)	28.8 ± 7.1	30.3 ± 7.1	1.1 ± 6.1

*Δ* changes in the parameters from preoperatively to 1 month postoperatively, *SD* Standard deviation, *TK* Thoracic kyphosis, *LL* Lumbar lordosis, *PT* Pelvic tilt, *SS* Sacral slope, *PI* Selvic incidence, *Cobb* Cobb angle of lumbar scoliosis, *SVA* Sagittal vertical axis, *ARO* Acetabular roof obliquity, *CE* Center edge angle

### Hip osteoarthritis evaluation by KL grade

**Fig. 3:**
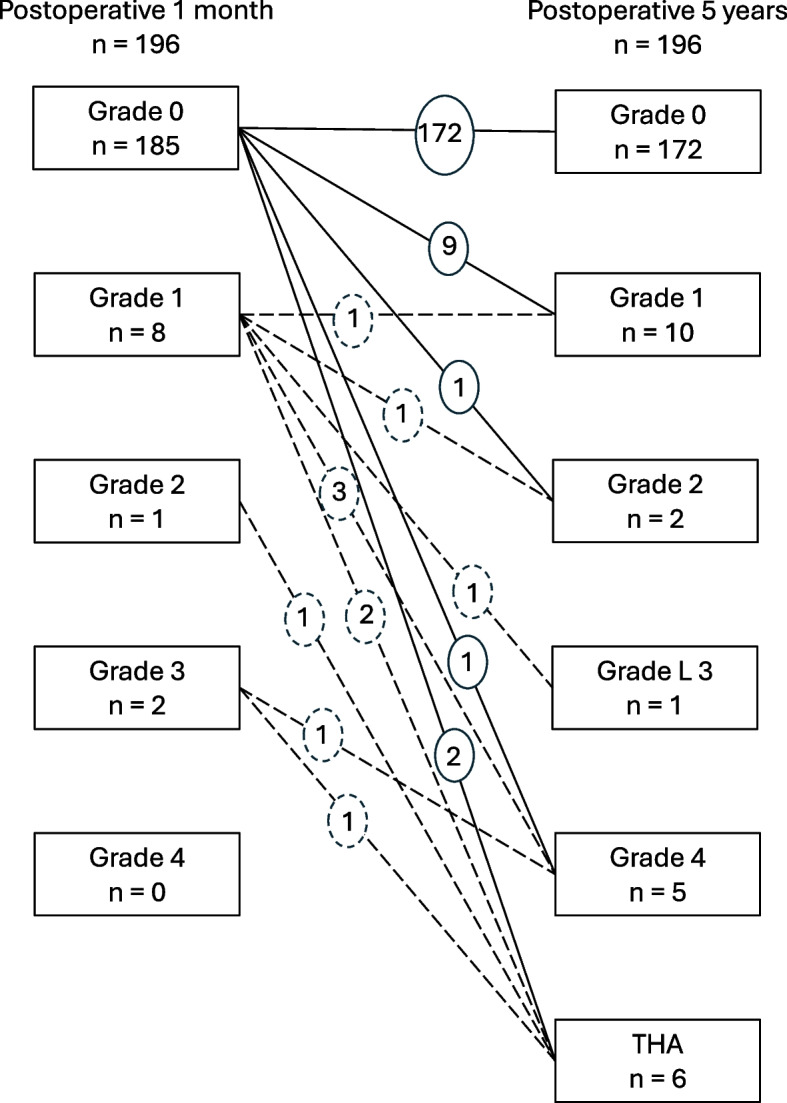
Change in KL grade from 1 month to 5 years after surgery. The solid lines represent the change from preoperative KL grade 0, and the dotted lines represent the change from preoperative KL grade 1–3. KL, Kellgren and Lawrence

KL grade at 1 month postoperatively was grade 0 in 185 joints, grade 1 in eight joints, grade 2 in one joint, grade 3 in two joints and grade 4 in none of the joints. KL grade at 5 years postoperatively was grade 0 in 172 joints, grade 1 in 10 joints, grade 2 in two joints, grade 3 in one joint and grade 4 in five joints (six joints underwent THA before the 5-year follow-up). Details of KL grade change from postoperative 1 month to 5 years are shown in Fig. 3. Finally, KL grade progression was observed in 23 joints. For overall analysis, the 23 joints with KL grade progression during the 5-year postoperative period were named group P, and the 173 joints with non-progression of KL grade were named group N.

We excluded cases with KL grade 1–3 at 1 month postoperatively and performed a limited analysis using 185 cases with KL grade 0. For this limited analysis, the 13 joints in which KL grade progressed from grade 0 were named Group P-0, and the 172 joints in which KL grade did not progress from grade 0 named group N-0.

### Comparison of parameters between groups

A comparison of demographic and 1-month postoperative radiographic parameters is shown in Table 2, and a comparison of changes in the parameters from preoperatively to 1 month postoperatively is shown in Table 3. Comparing group P and group N, there were significant differences in age (*p* = 0.035) and the ratio of patients with lumbosacral fixation (*p* = 0.045) in demographic data. Spinopelvic radiographic parameters did not differ significantly, although there were significant differences in hip joint parameters, such as ARO (*p* < 0.01) and CE (*p* < 0.01). Comparison of the changes in parameters between the two groups showed that the decreases in absolute values of ΔPI (*p* < 0.01) were significantly higher in group P than group N.

**Table 2:** Demographic and radiographic data 1 month postoperatively by group

	Group P (Mean ± SD)	Group N (Mean ± SD)	*p* value	Group P-0 (Mean ± SD)	Group N-0 (Mean ± SD)	*p* value
Age (years)	69.6 ± 1.8	65.1 ± 0.7	0.035	69 ± 2.5	65.1 ± 0.7	0.196
Female (%)	95.7	80.9	0.137	92.3	80.8	0.468
BMI (kg/m^2^)	23.8 ± 5	22.5 ± 3.3	0.4	25.2 ± 1	22.5 ± 0.3	0.033
No. of levels fused	7.8 ± 0.4	7.1 ± 0.2	0.064	7.5 ± 0.6	7.1 ± 0.2	0.271
LSF rate (%), n	91.3, 21/23	74, 128/173	0.045	84.6, 11/13	73.8, 127/172	0.366
TK (˚)	23.6 ± 2.5	27.9 ± 0.9	0.208	27.5 ± 3.4	27.8 ± 0.9	0.874
LL (˚)	38.8 ± 2.5	41.4 ± 0.9	0.49	37.8 ± 3.3	41.2 ± 0.9	0.43
PT (˚)	24.8 ± 1.9	22.6 ± 0.7	0.343	27.9 ± 2.6	22.5 ± 0.7	0.094
SS (˚)	28.1 ± 1.6	27.4 ± 0.6	0.6	26.5 ± 2.2	27.3 ± 0.6	0.651
PI (˚)	52 ± 2.2	50.2 ± 0.8	0.456	54 ± 3	50 ± 0.8	0.306
PI-LL (˚)	13.1 ± 12.9	8.7 ± 12.7	0.072	16.2 ± 11.5	8.8 ± 12.9	0.029
Cobb (˚)	13.9 ± 9.2	13.4 ± 10.1	0.837	11.2 ± 9.1	13.7 ± 10.3	0.441
SVA (mm)	32.8 ± 7.3	25.1 ± 2.7	0.119	38.6 ± 9.8	24.9 ± 2.7	0.074
ARO (˚)	12.4 ± 1.1	7.1 ± 0.4	< 0.01	9.6 ± 1.4	7.2 ± 0.4	0.273
CE (˚)	25.4 ± 9.2	30.9 ± 6.5	< 0.01	27.6 ± 1.9	30.9 ± 0.5	0.211

*SD* standard deviation, *LSF* lumbosacral fusion, *TK* thoracic kyphosis, *LL* lumbar lordosis, *PT* pelvic tilt, *SS* sacral slope, *PI* pelvic incidence, *Cobb* Cobb angle of lumbar scoliosis, *SVA* sagittal vertical axis, *ARO* acetabular roof obliquity, *CE* center edge angle

**Table 3:** Changes in the parameters from preoperatively to 1 month postoperatively by group

	Group P (Mean ± SD)	Group N (Mean ± SD)	*p* value	Group P-0 (Mean ± SD	Group N-0 (Mean ± SD)	*p* value1
ΔTK (˚)	11.6 ± 2.3	10.7 ± 0.8	0.792	11.4 ± 3.1	10.6 ± 0.8	0.959
ΔLL (˚)	33.3 ± 4.1	35 ± 5.5	0.66	34 ± 5.5	34.5 ± 1.5	0.836
ΔPT (˚)	-15 ± 1.9	-11 ± 1.7	0.083	-14.6 ± 2.6	-11 ± 0.7	0.236
ΔSS (˚)	10.4 ± 1.8	11.3 ± 0.7	0.623	8.9 ± 2.4	11.3 ± 0.7	0.429
ΔPI (˚)	-5.4 ± 5.9	0.2 ± 0.3	< 0.01	-7 ± 1.2	0.2 ± 0.3	< 0.01
ΔPI-LL (˚)	-38.8 ± 20.5	-34.8 ± 20.2	0.468	-41 ± 19.7	-34.3 ± 20.1	0.34
ΔCobb (˚)	-11.4 ± 10.4	-15.5 ± 11.8	0.078	-7.2 ± 4.6	-15.2 ± 11.1	< 0.01
ΔSVA (mm)	-69.6 ± 14.1	-82.1 ± 5.1	0.512	-70.1 ± 19.2	-81.8 ± 5.3	0.662

*Δ* changes in the parameters from preoperatively to 1 month postoperatively, *SD* standard deviation, *TK* thoracic kyphosis, *LL* lumbar lordosis, *PT* pelvic tilt, *SS* sacral slope, *PI* pelvic incidence, *Cobb* Cobb angle of lumbar scoliosis, *SVA* sagittal vertical axis

Comparing group P-0 and group N-0, there was a significant difference in BMI (*p* = 0.033) and PI – LL (*p* = 0.029). Comparison of the changes in parameters between the two groups showed that the decreases in absolute values of ΔPI (*p* < 0.01) was significantly higher and ΔCobb (*p* < 0.01) was significantly lower in group P-0 than group N-0.

Factors associated with KL grade progression.

In the comparison between group P and group N as overall analysis, there were significant differences in age, the ratio of patients with lumbosacral fixation, ARO, CE, and ΔPI, so multiple logistic regression analysis was performed using these as explanatory variables and the progression of KL grade as the objective variable. As a result, age (odds ratio (OR): 1.088, 95% CI: 1.006–1.178, *p* = 0.02), ARO (OR: 1.177, 95% CI: 1.017–1.36, *p* = 0.028), and ΔPI (OR: 0.79, 95% CI: 0.687–0.908, *p* < 0.001) were factors significantly involved in KL grade progression (Table 4).

**Table 4:** Factors associated with KL grade progression by multiple logistic regression analysis in overall analysis

	Odds ratio	95% CI	*p* value
Age	1.088	1.006–1.178	0.02
LSF rate	1.51	0.733–1.518	0.775
ARO	1.177	1.017–1.36	0.028
CE	0.963	0.928–1.162	0.507
ΔPI	0.79	0.687–0.908	< 0.001

*KL* Kellgren and Lawrence, *CI* confidence interval, *LSF* lumbosacral fusion, *ARO* acetabular roof obliquity, *CE* center edge angle, *Δ* changes in the parameters from preoperatively to 1 month postoperatively, *PI* pelvic incidence

In the comparison between group P-0 and group N-0 as limited analysis, there were significant differences in BMI, PI-LL, ΔPI, and ΔCobb, so multiple logistic regression analysis was performed using these as explanatory variables. As a result, PI-LL (OR: 1.058, 95% CI: 1.001–1.117, *p* = 0.04), ΔPI (OR: 0.785, 95% CI: 0.649–0.951, *p* < 0.001), and ΔCobb (OR: 1.127, 95% CI: 1.012–1.253, *p* = 0.009) were factors significantly involved in KL grade progression (Table 5).

**Table 5:** Factors associated with KL grade progression by multiple logistic regression analysis in limited analysis

	Odds ratio	95% CI	*p* value
BMI	1.067	0.935–1.259	0.44
PI-LL	1.058	1.001–1.117	0.04
ΔPI	0.785	0.649–0.951	< 0.001
ΔCobb	1.127	1.012–1.253	0.009

*KL* Kellgren and Lawrence, *CI* confidence interval, *BMI* body mass index, *PI* pelvic incidence, *LL* lumbar lordosis, *Δ* changes in the parameters from preoperatively to 1 month postoperatively

## Discussion

**Fig. 4:**
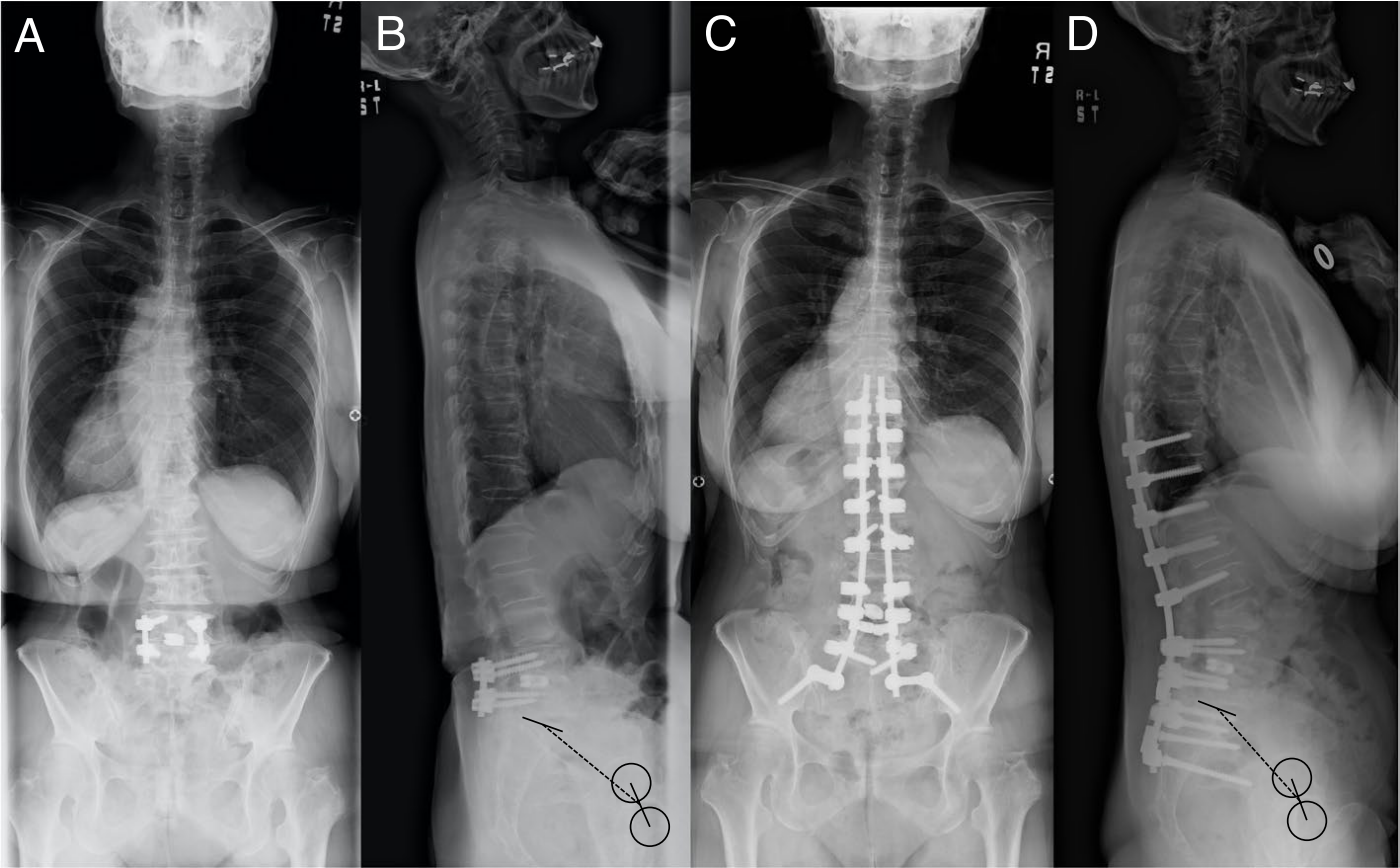
Radiographs of a 75 year-old female adult spinal deformity. Preoperative coronal and sagittal radiograph (SS = 21˚, PT = 50˚, PI = 70˚, LL = 29˚) (**A** and **B**, respectively). One-month postoperative coronal and sagittal radiograph (SS = 20˚, PT = 40˚, PI = 61˚, LL = 40˚) (**C** and **D**, respectively). The dotted line connects the midpoint of the S1 endplate and the midpoint of the line connecting the centers of the femoral heads bilaterally. Note the postoperative PI decreased by 9˚ compared to the preoperative value (**D**). SS, sacral slope; PT, pelvic tilt; PI, pelvic incidence; LL, lumbar lordosis

**Fig. 5:**
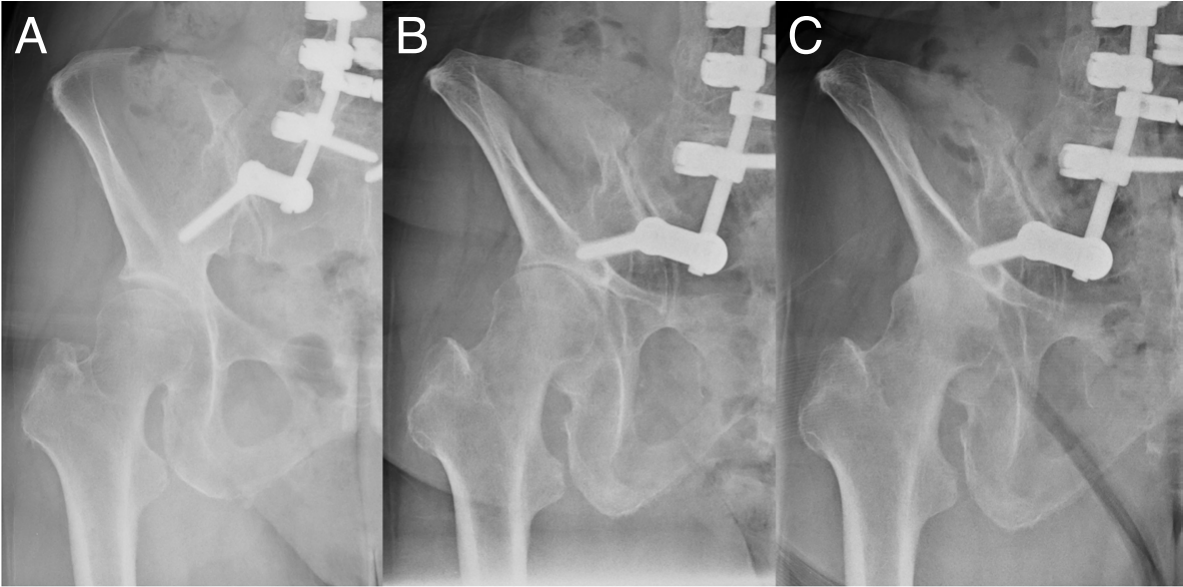
Left hip joint posteroanterior radiographs of the patient shown in Fig. 2. There were no visible features suggesting hip osteoarthritis at one month after surgery (ARO = 18˚, CE = 28˚) (**A**), although the osteoarthritis had progressed to KL grade 3 at 36 months after surgery (**B**), and KL grade 4 at 42 months after surgery (**C**). ARO, acetabular roof obliquity; CE, central edge angle; KL, Kellgren and Lawrence

In both the overall and limited analyzes of this study, ΔPI was the only parameter identified as influencing the progression of hip osteoarthritis after spinal fusion surgery in patients with ASD (Figs. 4 and 5). No previous studies have verified the preoperative to postoperative change in PI as a risk factor for hip osteoarthritis progression. One study that explored the correlation between hip pain and spinopelvic parameters reported that preoperative to postoperative change in PI did not correlate significantly with postoperative hip pain [12]. However, it would be unwise to generalize the result because the outcome of that study was hip pain, and not osteoarthritis progression. Our study demonstrated that preoperative to postoperative decrease in PI correlates significantly with KL grade progression. When assessing global sagittal alignment, PI is usually treated as an individual-specific pelvic morphological parameter with a fixed value [13, 14]. However, several reports have documented PI decrease on the operating table and after surgery with pelvic instrumentation [15–17]. Ohya et al. examined PI change in the prone position on the operating table compared with the standing position and reported that 12.5% of patients showed a decrease in PI of > 10˚ [15]. Referring to a biomechanical study, they suggested the possible mechanism as involving pressure on the apex of the sacrum in the prone position, leading to counternutation of the sacroiliac joint (decrease in the sacral tilt relative to the ilium) [18]. Oba et al. reported that PI decreased significantly (-3.3˚) after ASD surgery using iliac screws [16]. Studies of patients undergoing ASD surgery with S2 alar-iliac (S2AI) screws also demonstrated similar decreases in PI after surgery; Tseng et al. and Wai et al. reported PI decrease in 55% and 22% of patients respectively [17, 19]. These studies of surgery with pelvic instrumentation explain the mechanism of PI decrease by preexisting sacroiliac joint laxity [17], prone positioning with hip extension on the operating table that caused sacroiliac joint movement [15], cantilever sagittal correction technique to induce ilium anteversion relative to sacrum [16]. The current study showed similar results as mentioned above, with a decrease in PI in 46% (31/68) of patients with sacroiliac joint fixation by iliac screws.

We believe that decrease in PI after ASD surgery contributes to hip osteoarthritis progression in two ways. First, is the loss of ability of the hip joint to absorb mechanical load. As mentioned above, patients with a decrease in PI after surgery likely have preexisting sacroiliac joint laxity, which might help to absorb mechanical load on the hip joint. Development of increased stress at mobile segments adjacent to the spinal fusion area have been previously amply reported [1–4]. Recently, Kozaki et al. proposed the concept of adjacent segment disease in the hip joint as a complication of spinal fusion, including sacroiliac joint fixation [3]. Hence, it is reasonable to suppose that a large decrease in PI after ASD surgery is a risk factor for hip osteoarthritis progression. Second, decrease in PI implies the occurrence of counternutation motion at the sacroiliac joint [18], i.e. anteversion of the ilium relative to the sacrum. Anteversion of the ilium alters acetabular coverage of the femoral head, leading to an increase in anterior coverage. However, several studies pointed out that increasing anterior femoral head coverage by the acetabulum potentially increases the risk of femoroacetabular impingement, leading to hip osteoarthritis [9, 10, 20]. It is also important to note the relationship between femoroacetabular impingement and pelvic kinematics. Physiological standing-to-chair sitting motion causes decrease in lumbar lordosis, pelvic retroversion and hip joint flexion [21, 22]. Riviere et al. proposed that insufficient pelvic retroversion when moving from the standing to sitting position is associated with the risk of anterior femoroacetabular impingement [10]. Naturally, spinal fusion surgery with sacroiliac joint fixation limits pelvic retroversion when changing from standing to sitting, which is a risk factor for hip osteoarthritis progression [3, 5]. Moreover, studies of kinematics from siting to standing indicated that the pelvis gradually rotates more anteriorly before the seat-off phase than in the upright sitting position [23]. Consequently, it seems reasonable that spinopelvic fixation with pelvic anteversion caused by counternutation is a risk factor for femoroacetabular impingement leading to hip osteoarthritis.

ARO and age were selected as risk factors for hip osteoarthritis progression in the overall analysis but not in the limited analysis. ARO and age are considered as factors involved in the natural progression of hip osteoarthritis in the general population [24–28]. The fact that ARO and age were identified as risk factors in only overall analysis that included pre-existing hip osteoarthritis is a result similar to that of the general population, rather than an effect of fusion surgery.

Opinions differ as to the effect of number of fixation segments and lumbosacral fusion as risk factors of hip osteoarthritis progression. Kozaki et al. investigated the risk factors by including 118 cases consisting of 64 sacroiliac joint fixations and a mean of 8.1 fixation segments, and concluded that sacroiliac joint fixation was the only significant risk factor, while number of fixation segments was not [3]. Kawai et al. investigated the risk factors by including 205 cases consisting of a relatively large number of short fusions (i.e. single segment fixation: 88 cases, 2–3 segment fixation: 64 cases, 4–6 segments fixation: 27 cases and over 6 segments: 26 cases) [6]. They concluded that the number of fixation segments was the only significant risk factor for hip osteoarthritis progression, although lumbosacral fusion was not included in the multivariate model. The current study was similar to the study of Kozaki et al., in that the number of fixation segments was relatively long (i.e. mean: 7.2, range: 3–14), but was not a significant risk factor for hip osteoarthritis. A possible reason for this observation is that both studies did not include short fusion cases (i.e. < 3 segments). Studies including more short fusion cases may be necessary to rigorously investigate the relationship between the number of fixation segments and osteoarthritis progression. On the other hand, in the current study, lumbosacral fusion was indicated as a significant risk factor by a comparative study in overall analysis but was not a significant factor by multiple logistic regression analysis. This could be because the majority of participants (76%) in our study were treated with lumbosacral fusion.

In the limited analysis, a large PI-LL was selected as a risk for osteoarthritis progression. When the pelvis tilts posteriorly as a compensatory action for the mismatch between PI and LL, the anterior coverage of the femoral head by the acetabulum decreases, which may be a mechanism contributing to hip osteoarthritis. However, in the results of this study, although PT was greater in Group P-0 than in Group N-0, the difference was not significant, so we cannot make a definite statement about the contribution of compensation by hip joint to the progression of osteoarthritis. An analytical method that categorizes sagittal global alignment and compensation using the values ​​of SVA, PI-LL, and PT may be effective in explaining this mechanism in the future [29, 30]. Although less correction of the Cobb angle was selected as a risk factor for osteoarthritis progression, it should be noted that there was no difference in the postoperative Cobb angle between P-0 group and N-0 group. We compared the preoperative Cobb angles with the assumption that the reason for the smaller amount of correction angle in the P-0 group was that the preoperative Cobb angle of the P-0 group was smaller than that of the N-0 group. As a result, preoperative Cobb angle in the P-0 group was significantly smaller than the Cobb angle in the N-0 group (18.3˚ vs 28.6˚, *p* = 0.03). The absence of major coronal deformity may be one of the risk factors for developing hip osteoarthritis after ASD surgery, but this is only an indirect speculation, and a final conclusion cannot be drawn in this study.

This study had certain limitations. First, since we did not use the value of joint space width to evaluate hip osteoarthritis progression, we could not quantitatively evaluate the rate of joint space narrowing. However, the long follow-up period in this study (minimum 5 years) seems sufficient for developing radiological changes in the hip joint that can be determined by KL grade. A study about validation of the definition of hip osteoarthritis concluded that, in epidemiological studies, radiological hip osteoarthritis might be best defined by the KL system [31]. Second, we could not evaluate the other reported risk factors, such as S2AI screw loosening [14]. Though we used iliac screws instead of S2AI to reinforce S1 screws, the current study did not evaluate S2AI loosening as a preventive factor for hip osteoarthritis. There would likely be differences in the state of postoperative loosening between S2AI and iliac screws [32]. Future studies comparing S2AI and iliac screws as risk factors for hip osteoarthritis are necessary. Third, although the majority of participants in our study (76%) were treated with lumbosacral fusion, the LIV of cases without lumbosacral fusion varied in levels from L3 to L5, leading to potential bias. Fourth, we have not fully evaluated systematic joint degeneration in our subjects. Sacroiliac joint laxity, which was thought to be the cause of decrease in PI, may be one of symptoms of systematic joint degeneration, including hip joint. Fifth, the number of joints with osteoarthritis progression is relatively small, so there is concern that the number is insufficient to use as the dependent variable for logistic regression analysis.

## Conclusions

Both the overall and limited analyzes of this study identified preoperative to postoperative change in PI as parameters affecting the hip osteoarthritis progression after spinal fusion surgery in patients with ASD. Because sacroiliac joint laxity was thought to be the cause of decrease in PI, patients with this risk factor should be carefully followed for possible hip osteoarthritis progression.

## Data Availability

The data used and/or analyzed during this study are available from the corresponding author on reasonable request.

## Abbreviations

ARO: Acetabular roof obliquity
CE: Center edge angle
KL: Kellgren and Lawrence
PI: Pelvic incidence
ASD: Adult spinal deformity
LL: Lumbar lordosis
PT: Pelvic tilt
SS: Sacral slope
TK: Thoracic kyphosis
SVA: Sagittal vertical axis
THA: Total hip arthroplasty
S2AI: S2 alar-iliac
